# Reproductive Health Risks Associated with Occupational and Environmental Exposure to Pesticides

**DOI:** 10.3390/ijerph18126576

**Published:** 2021-06-18

**Authors:** Aleksandra Fucic, Radu C. Duca, Karen S. Galea, Tihana Maric, Kelly Garcia, Michael S. Bloom, Helle R. Andersen, John E. Vena

**Affiliations:** 1 Institute for Medical Research and Occupational Health, 10000 Zagreb, Croatia; 2 Unit Environmental Hygiene and Human Biological Monitoring, Department of Health Protection, National Health Laboratory, L-3555 Dudelange, Luxembourg; Radu.DUCA@lns.etat.lu; 3 Centre for Environment and Health, KU Leuven, 3001 Leuven, Belgium; 4 Institute of Occupational Medicine, Edinburgh EH14 4AP, UK; karen.Galea@iom-world.org; 5 Medical School, University of Zagreb, 10000 Zagreb, Croatia; tihana.maric@mef.hr; 6 Department of Global and Community Health, George Mason University, Fairfax, VA 22030, USA; kgarcia6@gmu.edu (K.G.); mbloom22@gmu.edu (M.S.B.); 7 Department of Public Health, University of Southern Denmark, DK-5000 Odense C, Denmark; HRAndersen@health.sdu.dk; 8 Department of Public Health Sciences, Medical University of South Carolina, Charleston, SC 29425, USA; Vena@musc.edu

**Keywords:** male, female, infertility, transplacental

## Abstract

A marked reduction in fertility and an increase in adverse reproductive outcomes during the last few decades have been associated with occupational and environmental chemical exposures. Exposure to different types of pesticides may increase the risks of chronic diseases, such as diabetes, cancer, and neurodegenerative disease, but also of reduced fertility and birth defects. Both occupational and environmental exposures to pesticides are important, as many are endocrine disruptors, which means that even very low-dose exposure levels may have measurable biological effects. The aim of this review was to summarize the knowledge collected between 2000 and 2020, to highlight new findings, and to further interpret the mechanisms that may associate pesticides with infertility, abnormal sexual maturation, and pregnancy complications associated with occupational, environmental and transplacental exposures. A summary of current pesticide production and usage legislation is also included in order to elucidate the potential impact on exposure profile differences between countries, which may inform prevention measures. Recommendations for the medical surveillance of occupationally exposed populations, which should be facilitated by the biomonitoring of reduced fertility, is also discussed.

## 1. Introduction

Many studies have investigated the effects of pesticides on male and female fertility. Similar to other agents present in the environment, exposure to pesticides may occur in occupational settings, through water consumption and dietary exposures, and from agricultural or gardening activities. Additionally, exposure may occur in populations residing next to crop and other agricultural fields, via sprayed pesticides spread by wind. The duration and timing of exposure to pesticides is likely to play a significant role in the severity of associated fertility disturbances [1].

Global pesticide use has increased over the past 30 years, with approximately 4 million tons of active ingredients consistently applied annually in recent years (FAOSTAT, 2020, http://www.fao.org/faostat/en/#data/RP/visualize last accessed on 13 April 2021). The United Nations Food and Agricultural Organization (http://www.fao.org/agriculture/crops/obsolete-pesticides/why-problem/pesticide-bans/en/, accessed on 19 April 2021) suggests that more than 20% of obsolete pesticide stockpiles consist of persistent organic pollutants (POPs), with some stockpiled pesticides being nearly 30 years old. They also suggest that there are instances where highly hazardous pesticides, which are not permitted for use in industrialized countries, are exported to developing countries [2].

The Pesticide Action Network International (PAN) reports that one or more of the 162 countries included in their consolidated list have banned a total of 460 active pesticide ingredients or groups of active ingredients regarded as “currently in use” in the global market, i.e., not considered to be obsolete (2021, PAN, 5th Edition, March 2021. Available from http://pan-international.org/pan-international-consolidated-list-of-banned-pesticides/, last accessed on 19 March 2021). The EU and UK were noted as the polities with the most known bans, with 175 banned and 208 specifically “not approved” pesticides, which are Highly Hazardous Pesticides according to their criteria. Switzerland (*n* = 140), Brazil (*n* = 131), Egypt (*n* = 76), Saudi Arabia (*n* = 73), Indonesia (*n* = 61), Cambodia (*n* = 60), India (*n* = 55), Mauritania (*n*= 52), Palestine (*n* = 52) and China (*n* = 51) were also highlighted as having high numbers of banned pesticides [3].

In 2019, Donley highlighted that whilst the PAN database is comprehensive and updated regularly, it does not include complete pesticide status data for the US, and it does not separate voluntary pesticide cancellation from non-voluntary cancellation in the US and EU [4]. The Rain Forest Alliance UTZ certification program developed a list of banned pesticides and a pesticides watchlist in 2015 (2015 UTZ list of banned pesticides and pesticides watch list. Version 1. Available from https://utz.org/?attachment_id=3272, accessed on 23 March 2021). The pesticides listed as reproductive toxicants are as follows: azafenidin, benomyl, borax, disodium tetraborate decahydrate, boric acid, dinocap, dinoterb, ethylene thiourea, fluazifop-butyl, flumioxazin, flusilazole, linuron, nitrobenzene, silafluofen, tridemorph, vinclozolin and warfarin [5].

Pesticides, including legacy organochlorine pesticides (OCPs), such as dichlorodiphenyltrichloroethane (DDT), hexachlorobenzene (HCB), and chlordane, as well as contemporary organophosphate-based agents, pyrethroids, triazines and others, may have a deleterious impact on human fertility. Thus, the International Federation of Gynecology and Obstetrics (FIGO) [6], the American College of Obstetrics and Gynecology (ACOG), and the American Society for Reproductive Medicine (ASRM) [7] have called for greater attention to accumulating evidence that non-occupational, or “background”, exposures to environmental pollutants, including pesticides, may be reproductive toxicants. At high doses, experimental evidence suggests that pesticide exposure modifies endocrine function, disrupting gonadotropin, thyroid, and sex-steroid hormone signaling [8,9], and induces oxidative stress [10,11], altering sexual development, fecundity and maintenance of pregnancy. However, human data remain controversial as regards the lower background levels of environmental pollutants, including pesticides, to which much of the general population is exposed continuously [12,13,14,15].

As the western world has faced a significant reduction in fertility during the last few decades [16,17,18,19], the aim of this comprehensive review is to update current knowledge on the association between environmental and occupational exposure to pesticides and fertility disturbances in men and women, although without estimating associations or assessing evidence of causality. This review also includes reproductive health risks after exposures to pesticides across the life-span, from intrauterine development, to childhood, to adulthood.

## 2. Material and Methods

Published studies to be included in this review were required to fulfill the following criteria: (a) full length papers reporting original data in a peer-reviewed journal; (b) self-reporting exposure and/or measured levels of metabolites in blood or urine; (c) minimum of 20 subjects and a corresponding referent group; (d) English language, and (e) published between and including the years 2000 and 2020. We also consulted relevant reviews. At all stages of the review process (title, abstract and full text), those articles considered to not fulfil the inclusion criteria were excluded. One case report and one commentary were excluded. The literature databases PubMed and Scopus were searched using the following search terms and strings: 

infertility + pesticide + exposure, abortion + pesticide + exposure; IVF + pesticide + exposure; pregnancy + pesticide + exposure, ovulation + woman + pesticides, and menarche + pesticides for female fertility; and sperm + pesticide + exposure and sperm + morphology + pesticide; pesticide(s) OR active ingredient + banned + withdrawn; pesticide + sperm; pesticide + transplacental; pesticide + intrauterine; pesticide + prenatal exposure +testosterone; prenatal + pesticide + exposure + infertility; prenatal + pesticide + exposure + semen; prenatal + fungicide + exposure + newborn; in utero + pesticide + newborn; in utero + pesticide + testosterone; in utero + pesticide + fertility; in utero + pesticide + estradiol; in utero + pesticide + semen; in utero + pesticide + puberty

**Figure 1:**
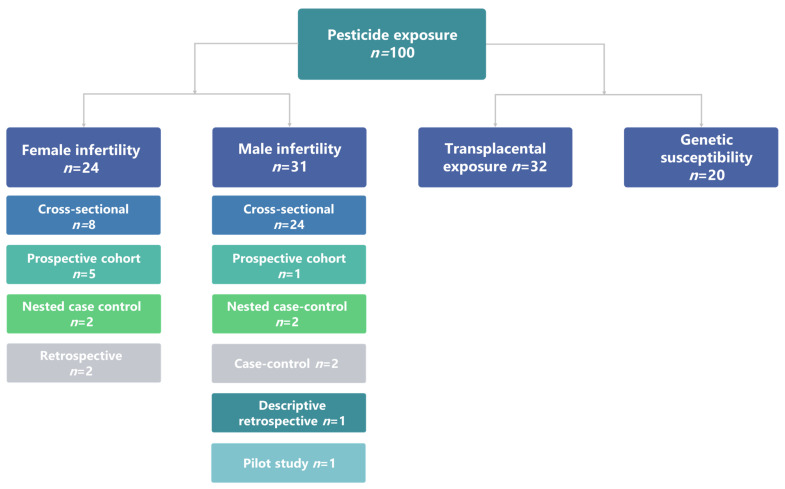
Graphical presentation of selected papers included in review article.

A schematic presentation of the scheme reporting the literature retrieval and selection strategy is shown in Figure 1. 

After the searches were complete, the titles and, where available, abstracts were screened by section authors to identify studies of relevance in accordance with the inclusion criteria. A conservative strategy was adopted, in which all potentially relevant citations were retained for full-text scanning. Following the screening of the titles and abstracts, copies of full papers were obtained for full reviewing. At least two authors read the full text of the selected papers for each section. After consensus was derived on the interpretation of the results, each author contributed to manuscript preparation.

Figure 1 gives insight into the numbers and types of papers included. The selection process resulted in a total of 107 articles, which were used in our review of associations between fertility outcomes, occupational, environmental, and transplacental exposure to pesticides, and age- or sex-specific susceptibility. 

The quality of the included articles was not individually assessed. This review focused on describing recent advances and summarizing fertility, sexual maturation and pregnancy complications associated with occupational, environmental and transplacental exposures to pesticides, rather than a formal review of the study quality or possible bias in the included epidemiological studies [20].

## 3. Results 

### 3.1. Female Fertility

The quantitative results of studies and the associated statistical significance are reported in Table 1.

#### 3.1.1. Time to Pregnancy and Infertility

The ability to conceive a pregnancy is frequently measured as time to pregnancy (TTP), which is the number of menstrual cycles with unprotected heterosexual intercourse to conceive a pregnancy without assistance [21,22]. TTP is often expressed as fecundability odds ratio (FOR), the conditional odds for conceiving in a given cycle (i.e., month), or as clinical infertility, which is more than 12 months of trying without a pregnancy [23]. 

Environmental Exposures

Studies Using Direct Exposure Assessment/Biomonitoring Strategies

In a retrospective study from Sweden, 22 POPs, including 9 OCPs and 12 non-pesticide polychlorinated biphenyls (PCBs), were measured in 1st trimester blood specimens (~10 weeks gestation) from 818 women with pregnancies in 2007–2010, and these were related to self-reported TTP [24]. The women were 18–43 years old. There was no association of individual serum OCPs with TTP, although 3rd quartile serum hexachlorobenzene (HCB) was associated with greater odds of infertility, adjusted for confounding. However, greater serum HCB was associated with longer TTP among younger women (<29 years) who had used combined oral contraceptives (COCs). In contrast, the 2nd and 4th quartiles of dichlorodiphenyldichloroethylene (p,p’-DDE), a persistent metabolite of DDT, were associated with longer TTP among older women (≥29 years) who had used COCs. To accommodate strong pairwise correlations, the authors implemented weighted quantile sum regression, an approach that allows one to estimate a single weighted exposure index of all 22 measured POPs [25]. Although greater overall POP exposure was associated with longer TTP among older women without use of COCs, important associations were limited to PCBs and no OCPs were identified as important. However, each quartile of overall POP exposure was also associated with greater odds of infertility among older women without the use of COCs, and 8.0% of the association was attributed to trans-nonachlor, a persistent lipophilic component of chlordane. 

In a prospective study from China, six organophosphate (OP) and three pyrethroid pesticide metabolites were measured in preconception urine from 615 women 24–44 years of age from the general population in 2013–2015 [26]. Women were followed prospectively until 12 months of trying or a pregnancy, including the time spent trying to conceive before study enrollment. Women in the highest quartile of urinary diethylthiophosphate (DETP) level, an OP insecticide metabolite, had longer TTP and greater odds of infertility than women in the lowest quartile of urinary DETP adjusted for confounding although without a linear trend. Women in the 4th quartile of urinary 3-phenoxybenzoic acid (3-PBA), a pyrethroid insecticide metabolite, also had longer TTP and greater infertility rates than women in the 1st quartile. 

Studies Using Indirect Exposure Assessment Strategies

A retrospective cohort investigation from Colombia found little evidence of an association between self-reported TTP, measured among 2592 women 15–54 years of age with a pregnancy in the past five years, and glyphosate exposure assigned to residential region in 2004–2005 [27]. Women residing in some areas with aerial glyphosate spraying programs had longer TTPs than women residing in a reference region without glyphosate spraying. However, residence in other regions without aerial glyphosate spraying were also associated with longer TTP, and there was no TTP difference between additional regions with and without glyphosate use.

#### 3.1.2. Assisted Reproduction

Women using assisted reproductive technologies, such as in vitro fertilization (IVF), are distinct in terms of the intensity of data collection and follow-up, presenting an unprecedented opportunity to observe early pregnancy events that are the critical, but usually unobserved, determinants of live birth, such as oocyte and embryo development [28]. These populations offer opportunities for disentangling “critical windows” of biologic vulnerability for infertility that are infeasible in populations conceiving spontaneously, including the collection of highly invasive biospecimens, such as ovarian follicular fluid. A recent narrative review suggested deleterious impacts on oocyte maturation, oocyte fertilization, embryo development, and ultimately live births associated with pesticide exposure [29]. 

Environmental Exposure

Studies Using Direct Exposure Assessment/Biomonitoring Strategies

A nested case–control study of three US infertility clinics measured serum HCB, p,p’-DDE, o,p’-DDE, p,p’-DDT and o,p’-DDT in association with IVF outcomes among 720 women with a mean (SD) age of 35.4 years (4.20) in 1994–2003 [30]. The serum OCPs positively correlated with measures in ovarian follicular fluid [31]. The authors reported a higher probability of a negative pregnancy test after embryo transfer among women with pre-cycle serum HCB in the 2nd quartile relative to the 1st quartile. Importantly, serum HCB levels were lower than those reported for US females in general. However, no association was detected with clinical pregnancy, or with serum DDE or DDT concentrations.

A small prospective investigation of 32 women, 28–42 years old, undergoing IVF at another US infertility clinic measured 45 POPs, including 43 PCBs, p,p’-DDT and p,p’-DDE, in ovarian follicular fluid [32]. DDE was detected in all follicular fluid specimens and both DDE and DDT were significantly higher among Asian women than white women. Greater DDE was associated with a lower probability to receive a mature oocyte, although a greater likelihood for live birth and DDT was associated with higher peak-estradiol.

Studies Using Indirect Exposure Assessment Strategies

A prospective investigation of 325 women undergoing treatment at a US infertility clinic, primarily IVF, reported lower likelihoods for pregnancy and live birth in association with greater consumption of high-pesticide residue fruits and vegetables, as reported on a study questionnaire taken in 2007–2016. The women were 18–45 years of age. The authors used the 2006–2015 Department of Agriculture Pesticide Data Program mean pesticide residue data to categorize reported foods as high-pesticide and low-pesticide residue items. Women in the highest quartile of high-pesticide fruit and vegetable consumption were significantly less likely to have a clinical pregnancy and a live birth than women in the lowest quartile of high-pesticide fruit and vegetable consumption. However, there was no association detected for consumption of low-pesticide residue fruits and vegetables [33].

#### 3.1.3. Ovarian Reserve and Hormone Levels

Environmental Exposure

Studies Using Direct Exposure Assessment/Biomonitoring Strategies

Jurewicz et al. in 2020 studied exposure to pyrethroid pesticides and ovarian reserve in 511 females, aged 25–39 years, attending an infertility clinic in Poland [34]. Greater urinary concentrations of 3-PBA were associated with decreased antral follicle count (*p* = 0.02) and levels of anti-mullerian hormone (AMH) (*p* = 0.03) and increased follicle-stimulating hormone (FSH) (*p* = 0.04). The authors concluded that synthetic pyrethroids may affect female ovarian reserve.

Studies Using Indirect Exposure Assessment Strategies

Whitworth et al. (2015) studied AMH in relation to indoor, primarily seasonal, DDT and pyrethroid spraying for malaria prevention among women 20–30 years of age in eight rural South African villages [35]. Women who reported indoor residual pyrethroid spraying had 25% lower AMH concentrations compared with women who reported no spraying, although there was only limited evidence for associations with DDT spraying. Prior reviews have suggested links between pesticide exposure and ovarian toxicity leading to female infertility, including premature ovarian insufficiency (POI) and polycystic ovary syndrome (PCOS), menstrual problems and altered sex steroid hormone synthesis [29,36,37,38,39], although the effects were likely dose-dependent and agent specific [40], and varied with the timing of the exposure [41].

#### 3.1.4. Spontaneous Fetal Loss (Miscarriage)

Occupational Exposure

Studies Using Indirect Exposure Assessment Strategies

Garry et al. studied exposures to pesticides and risk of pregnancy loss in US applicators (98% men) and their spouses (women) [42]. Women were 15 to >35 years of age at the time of pregnancy. Among the 695 participants, there was a modest but significant increase in risk (1.6-fold to 2-fold) of miscarriages and/or fetal loss occurring throughout the year in the spouses of applicators who used fungicides, with the greatest risks during pesticide application in spring. Women using pesticides experienced a greater risk for miscarriage as did women whose male partner used pesticides containing sulfonylurea, imidizoline, and a mixture of chlorophenoxy, sulfonylurea, and benozothiodiazole.

Naidoo et al. [43] examined cross-sectional associations of pesticide exposure with spontaneous miscarriage among 911 women 18–82 years of age, working in two distinct South African agricultural areas (i.e., the irrigation scheme and drylands). Spraying pesticides during the first three months of a pregnancy was associated with a greater prevalence of spontaneous miscarriage. Older (≥40 years of age) irrigation scheme women were more likely to report spontaneous miscarriage than younger women. An important limitation was that exposure was assessed by occupational history only. 

Rahimi et al. interviewed women working in greenhouses and married for at least one year, with 338 unexposed controls from nearby villages, in a cross-sectional study from southern Iran [44]. The women were 16–49 years of age. The greenhouse workers had a significantly (*p* = 0.002) greater number of spontaneous pregnancy losses before 20 weeks completed gestation (mean = 0.26) than the control women (mean = 0.18), although this is unadjusted for confounding factors or number of pregnancies. 

#### 3.1.5. Age at Menarche

Occupational Exposure

Studies Using Direct Exposure Assessment/Biomonitoring Strategies

In a study on 151 female offspring of US anglers 20–50 years of age, each 15 ug/L increase in back-extrapolated gestational DDE exposure was associated with one year earlier menarche (*p* = 0,04), adjusted for confounders [45]. However, the association became non-significant when controlling for estimated body size at menarche among a subsample of *n* = 102. 

Environmental Exposure

Studies Using Direct Exposure Assessment/Biomonitoring Strategies

A cross-sectional analysis of 305 girls aged 9–15 years old from Hangzhou, China, found that pyrethroid exposure may be associated with a greater prevalence of delayed pubertal onset [46]. This study analyzed urinary 3-PBA at the time of the interview, and this was detected in 89% of the participants. Using self-assessed Tanner stages, pubertal onset by the odds of breast stage 3, pubic hair stage 2 and self-reported menarche were associated with a greater urinary 3-PBA level.

A cross-sectional analysis of nulliparous and newly married Chinese women, 20–34 years of age, showed an earlier self-reported age at menarche in association with higher serum DDT concentrations [47]. Relative to those in the lowest DDT quartile, the adjusted mean age of menarche was approximately 1.11 years younger among those in the fourth quartile, with a significant linear trend. 

A nested case–control study found a weak, non-significant association of in utero exposure with the atrazine metabolite diaminochlorotriazine (DACT), measured in gestational maternal urine specimens (median = 12 weeks), and with early menarche [48]. The study was conducted within a larger prospective cohort, in which the offspring of 14,775 women were mailed an annual health questionnaire from 8 to 17 years of age. Maternal exposure was collected for 174 cases of early menarche (<ll.5 years of age) and 195 controls (>11.5 years of age). DACT was found to be the most frequently detected analyte (58% above LOD). This association became statistically significant in a subset of *n* = 85 that excluded girls with missing data.

#### 3.1.6. Menstrual Cycle Length

Environmental Exposure

Studies Using Direct Exposure Assessment/Biomonitoring Strategies

In a prospective cohort, the relationship between a mixture of POPs, menstrual cycle length and duration of bleeding among women who are attempting to become pregnant was investigated [49]. Select POPs were found to be associated with differences in menstruation. Women aged 18–44 years were recruited from a larger angler cohort study, residing in counties along Lakes Erie and Ontario. Eighty-three participants contributing to 447 cycles for analysis provided a blood sample at enrollment for the quantification of 76 PCBs and seven OCPs. A statistically significant three-day increase in cycle length was observed for women in the highest tertile of estrogenic PCBs relative to the lowest tertile. A significant reduction in bleeding (less than one day) was seen among women in the highest versus the lowest tertile of aromatic fungicide exposure.

In the aforementioned cross-sectional study of 466 newly married, nulliparous Chinese women aged 20–34 years [47], women in the fourth quartile for serum DDT (range: 41.6–113 ng/g; mean = 57.1 ng/g) exposure had 2.78 times higher odds of experiencing a short menstrual cycle (defined as ≤ 21 days) in the previous year relative to those in the lowest DDT quartile (range: 5.52–19.2 ng/g; mean = 13.5 ng/g). These odds became even stronger after the exclusion of women who drank alcohol, used oral contraceptives/intrauterine device (IUD), or who experienced a spontaneous or induced abortion. In contrast, there was no association between DDT and long menstrual cycles.

**Table 1:** Epidemiologic studies estimating associations between pesticide exposure and female fertility.

Study	Site	Design	Exposure	Duration of Exposure	Sample	Outcome	Results
Björvang et al., 2020 [24]	Sweden (environmental exposure)	Cross-sectional	Serum gestational POPs (9 OCPs, 10 PCBs and 3 PBDEs)	Long-term	*n* = 818 women with a pregnancy	TTP and clinical infertility	3rd Q vs. 1st Q HCB associated with: (1) infertility (OR = 2.25, 95%CI: 1.06, 4.78); (2) longer TTP among women < 29 years of age who used COCs. DDE associated with longer TTP among women ≥ 29 years who used COCs.
Bloom et al., 2017 [32]	United States (environmental exposure)	Prospective cohort	Ovarian follicular fluid organochlorines (2 OCPs and 43 PCBs)	Long-term	*n* = 32 women undergoing IVF	Measures of ovarian reserve and IVF outcomes	DDE associated with oocyte maturity (RR = 0.72; 95% CI: 0.60, 0.86) and live birth (RR = 3.65; 95% CI: 1.08, 12.31). DDT associated with E2 (β = 1.45, 95%CI: 0.92, 1.97)
Chiu et al., 2018 [33]	United States (environmental exposure)	Prospective cohort	Self-reported consumption of fruit and vegetable pesticide residues	Short-term (past 3 months)	*n* = 325 women undergoing IVF (541 cycles)	Pregnancy and live birth	High pesticide residue fruits and vegetables associated with lower probabilities for pregnancy (*p* = 0.04) and live birth (*p* = 0.02).
Garry et al., 2002 [42]	United States (Occupational exposure)	Cross-sectional	Self-reported application of herbicides, insecticides, fungicides and fumigants	Current and historic	*n* = 522 women with singleton pregnancies fathered by a licensed pesticide applicator	Pregnancy loss and menstrual function	Herbicide/insecticide/fungicide use vs. herbicide only use associated with pregnancy loss (OR = 1.64, 95%CI: 1.01, 2.67).
Hu et al., 2018 [26]	China (environmental exposure)	Prospective cohort	Urinary organophosphate (6) and pyrethroid (3) metabolites	Short-term	*n* = 615 women planning a pregnancy	TTP and clinical infertility	4th Q vs. 1st Q DETP associated with TTP (FOR = 0.68, 95%CI: 0.51, 0.92) and infertility (OR = 2.17, 95%CI: 1.19, 3.93); 4th Q vs. 1st Q 3-PBA associated with TTP (FOR = 0.72, 95%CI: 0.53, 0.98) and infertility (OR = 2.03, 95%CI: 1.10–3.74) in nulliparas.
Jurewicz et al., 2020 [34]	Poland (environmental exposure)	Cross-sectional	Urinary pyrethroid metabolites (4)	Short-term	*n* = 511 women undergoing infertility evaluation	Measures of ovarian reserve	3-PBA associated with AFC (β = −0.02 follicles, 95%CI: −0.06, −0.01), AMH (β = −0.04 ng/mL, 95%CI: −0.12, −0.04), FSH (β = 0.01 IU/L, 95%CI: 0.01, 0.25) per ng/mL.
Louis et al., 2011 [49]	United States (environmental exposure)	Prospective cohort	Serum organochlorines (7 OCPs and 76 PCBs)	Long-term	*n* = 83 women planning a pregnancy (447 cycles)	Menstrual function	3rd T vs. 1st T aromatic fungicides associated with days bleeding (β=−0.15 days, 95%CI: −0.29, −0.002).
Mahalingaiah, 2012 [30]	United States (environmental exposure)	Nested case control	Serum DDT, DDE and HCB	Long-term	*n* = 720 women undergoing IVF (827 cycles)	Failed implantation, pregnancy and pregnancy loss	4th Q vs. 1st Q HCB associated with failed implantation (OR = 2.32, 95%CI: 1.38, 3.90).
Naidoo et al., 2011 [43]	South Africa (occupational exposure)	Cross-sectional	Self-reported pesticide spraying	Short-term (during early pregnancy)	*n* = 887 farm workers with a pregnancy	Pregnancy loss	Spraying pesticides associated with greater risk of spontaneous loss (OR = 2.6, 95%CI: 1.6, 6.4).
Namulanda et al., 2017 [48]	England (environmental exposure)	Nested case control	Gestational urinary atrazine metabolites (6)	Short-term	*n* = 369 girls (174 cases, 195 controls)	Menarche (<11.5 years)	Diaminochlorotriazine associated with non-significant odds of early menarche (OR = 1.13, 95% CI: 0.82, 1.55).
Ouyang et al., 2005 [47]	China (environmental exposure)	Cross-sectional	Serum DDT and DDE	Long-term	*n* = 466 nulliparous textile workers	Menarche and menstrual function	DDT associated with: (1) age at menarche (−0.20 years, 95%CI: −0.28, −0.13 per 10 ng/g); (2) short menstrual cycle (<21 days) 4th Q vs. 1st Q (OR = 2.78, 95%CI 1.07, 7.14).
Rahimi et al., 2020 [44]	Iran (occupational exposure)	Cross-sectional	Self-reported workplace pesticide exposure	Current and historic	*n* = 645 women (308 exposed greenhouse workers and 337 unexposed housewives)	Pregnancy loss and infertility	Greenhouse workers had significantly greater prevalence of spontaneous loss (*p* = 0.019) and infertility (*p* = 0.003) than housewives.
Sanin et al., 2009 [27]	Colombia (environmental exposure)	Retrospective cohort	Aerial glyphosate spraying programs in 4 regions	Up to 12 months (while trying to conceive)	*n* = 2592 women with a pregnancy in prior 5 years	TTP	TTP greater in regions with glyphosate spraying than without (*p* < 0,01).
Vasiliu et al., 2004 [45]	United States (environmental exposure)	Retrospective cohort	Back extrapolated pregnancy serum DDE and PCBs	Long-term	*n* = 151 daughters of Lake Michigan anglers	Menarche and menstrual cycle	Extrapolated gestational DDE associated with age at menarche (β = −0.07 years; *p* = 0.038 per µg/L).
Whitworth et al., 2015 [35]	South Africa (environmental exposure)	Prospective cohort	Plasma DDT and DDE and indoor pesticide spraying	Short-term (seasonal)	*n* = 420 women from rural villages	Plasma AMH	Indoor pyrethroid spraying vs. no indoor spraying associated with AMH (−25%, 95%CI: −39%, −8%).
Ye et al., 2017 [46]	China (environmental exposure)	Cross-sectional	Urinary 3-PBA (a pyrethroid metabolite)	Short-term	*n* = 305 girls (9−15 years of age)	Self-assessed (with mother) sexual development (Tanner stages)	3-PBA associated with breast stage 3 (OR = 0.55, 95%CI: 0.31, 0.98), pubic hair stage 2 (OR = 0.56, 95%CI: 0.36, 0.98) and menarche (OR = 0.51, 95%CI: 0.28, 0.93).

Note: AFC, antral follicle count; AMH, anti-mullerian hormone; CI, confidence interval; COCs, combined oral contraceptives; DDE, dichlorodiphenyldichloroethane; DDT, dichlorodiphenyltrichloroethane; DETP, diethylthiophosphate; E2, estradiol; FSH, follicle-stimulating hormone; HCB, hexachlorobenzene; IVF, in vitro fertilization; OCPs, organochlorine pesticides; OR, odds ratio; PBDEs, polybrominated diphenyl ethers; PCBs, polychlorinated biphenyls; POPs, persistent organic pollutants; Q, quartile; RR, relative risk; T, tertile; TTP, time to pregnancy; 3-PBA, 3-phenoxybenzoic acid.

### 3.2. Male Infertility

Quantitatve results of the studies and the associated statistical significance are reported in Table 2.

Occupational Exposure

Studies Using Indirect Exposure Assessment/Biomonitoring Strategies

Occupational exposure to pesticides has been well-documented in several farm communities across the globe. A ten-year retrospective study of Cameroonian men seeking urology services showed that patients older than 50 years and farmers were the most susceptible to poorer semen quality. The researchers believed that this was due to higher pesticide exposure, which was not directly measured in the study, combined with high temperature [50]. Sperm analysis from farmers in Myanmar, who were occupationally exposed to different types of pesticides on a daily basis, showed decreased sperm counts during the growing period compared to non-growing periods. In the growing period, farmers were actively exposed to OP pesticides. Moreover, in the growing period, reproductive hormone levels differed significantly from levels measured in the non-growing period. It is important to stress that in this region men started working as farmers almost immediately after primary school and did not regularly wear protective equipment [51]. African researchers stated that the decreasing sperm count in African men may have been due to exposure to pesticides, especially dibromochloropropane among farmers in the late 1970s [52]. Iranian researchers also observed that farmworkers had a higher prevalence of primary male infertility than the general population, possibly due to their recurrent exposure to pesticides [53]. Young men from rural regions of Brazil also showed poorer sperm quality than men from the urban parts of the country. There was a lower percentage of normal sperm morphology in men using pesticides over longer period, along with higher sperm counts and lower LH levels. On the other hand, this study also showed that larger testicular volumes and longer anogenital distances were associated with maternal farming during pregnancy [54]. Farm worker exposure to pesticides that have been banned or strictly controlled in developed countries remains a concern in Venezuela. Pesticide-exposed farmers in Venezuela had a higher DNA fragmentation index and lower sperm quality than unexposed farmers, while 44% of the subjects had increased LH [55]. Another study showed lower semen volume, sperm motility, normal morphology percentage, seminal zinc concentration as well as testosterone and LH in men who were pesticide applicators. On the other hand, seminal pH, time of liquefaction, percentage of immature sperm and leukocyte concentration were higher in the same group compared to men who were not pesticide applicators. In this study, specific OP metabolites were not correlated with parameters of semen quality [56]. According to these studies, farm workers from rural regions are a group that are critically exposed to pesticides, and therefore the group with highest pesticide impact on infertility. Additionally, men from rural areas may exhibit disrupted quality of sperm parameters and lower serum testosterone and LH, which can potentially correlate with exposure to pesticides [57].

Studies Using Direct Exposure Assessment/Biomonitoring Strategies

The urinary metabolites of OPs were also tested in occupationally exposed Peruvian men through sprayer applicator usage. Decreased seminal plasma volume and increased pH were associated with higher levels of OP metabolites. More importantly, the exposure level and time of the exposure together showed a more profound effect on sperm quality [58]. Abamectin (ABM), used by Turkish farmers, was also correlated with lower sperm quality. Levels of ABM, measured in farmers’ plasma, were relatively high compared to a control group of men. Furthermore, the exposed farm workers had significantly decreased sperm motility and impaired sperm maturity markers, observed by measuring hyaluronic acid sperm binding, as well as higher percentages of aniline blue sperm and increased creatinine kinase-positive sperm [59]. Among male floriculture workers in Mexico, urinary concentrations of OPs were associated with increased serum concentrations of FSH and prolactin, and with decreased serum testosterone and inhibin B [60].

Environmental Exposure

Studies Using Direct Exposure Assessment/Biomonitoring Strategies

A recent study of reproductive-aged Chinese men associated pyrethroid metabolites in urine with semen quality. They found negative associations between 3-PBA and sperm morphology, as well as for trans-3-(2,2-Dichlorovinyl)–2,2-dimethylcyclopropane carboxylic acid (t-DCCA) levels and total sperm count [61]. Another study on non-occupational exposure to pyrethroids among Chinese men also showed significant correlations between higher quartiles of 3-PBA levels in urine and sperm concentration, motility and DNA integrity [62]. Urine samples from a healthy Japanese student population contained 3-PBA in 91% of the subjects, but a correlation with reproductive hormone levels was absent. Likewise, 3-PBA was not associated with semen quality in the same group of men [63,64]. These Japanese and Chinese studies reported contradictory results regarding correlations between similar 3-PBA urinary levels and semen quality; the main differences in the studies were the study groups—healthy students in the Japanese study and infertility patients in the Chinese study. Another study of Japanese men attending a fertility clinic showed that pyrethroid metabolites and dietary habits combined were associated with poorer sperm quality, with 3-PBA correlated with lower sperm motility [65]. Moreover, a Polish study on urinary metabolites of pyrethroids had similar outcomes as the Chinese study, also reporting an association between urinary pyrethroid metabolites and semen quality among men attending an infertility clinic. Abnormal sperm morphology was associated with higher levels of cis-3-(2,2-Dichlorovinyl)–2,2-dimethylcyclopropane carboxylic acid (c-DCCA), t-DCCA, and the sum of pyrethroid metabolites, while lower sperm concentration and testosterone levels were associated with t-DCCA [66]. A study by Meeker et al. also suggested an association between urinary 3-PBA and t-DCCA and sperm concentration, while c-DCCA and 3-PBA were associated with increased DNA damage [67]. Furthermore, men with higher c-DCCA and t-DCCA levels in urine had increased sex chromosome disomies compared to men with lower urinary levels. 3-PBA was also related to an increased risk of YY disomies. The mechanism behind these findings is not fully understood. Pyrethroid metabolites are rapidly excreted, but long-term exposure might negatively affect spermatogenesis and meiosis [68].

Pant et al. measured concentrations of POPs, specifically p, p’-DDE and lindane, in semen. Decreased semen quality and higher ROS production were correlated with seminal p, p’-DDE and lindane [69]. The serum of male partners in subfertile couples was analyzed for PCBs and p, p’-DDE in another study [70]. There was a negative dose–response relationship between PCB-138 and sperm concentration, motility and morphology; however, an association of sperm parameters and other PCBs with p, p’-DDE was not detected [70]. Norwegian researchers also investigated the impact of POPs on male reproductive health in men from southern and northern Norway. The authors hypothesized that men residing closer to the Arctic Circle would accumulate more POPs, specifically 2,2′4,4′,5,5′-hexachlorobiphenyl (CB-153) and p, p’-DDE. However, plasma levels of CB-153 did not differ significantly between men from the south and north, and p, p’-DDE levels were higher in the southern population. CB-153 and sex hormone-binding globulin (SHBG) were positively correlated. There was no association between sperm motility and CB-153 or p, p’-DDE, but a link was observed between sperm concentration and CB-153 in the south [71]. A population from the Faroe Islands was analyzed for correlations between p, p’-DDE and PCBs and sperm disomy, as this population has a history of high organochlorine exposure. Men with elevated exposure to p, p’-DDE and PCBs had higher rates of extra X chromosome and total sex-chromosome disomy. This study also included a birth cohort in which blood levels of p, p’-DDE and PCBs were measured in cord blood, at 14 years and in adulthood. Sex-chromosome disomy was associated with exposure to organochlorines at 14 years of age, which is the time for onset of puberty [72]. Secondary sex ratio (SSR) and exposure to POPs have also been studied in couples attending a fertility clinic, but there was no clear correlation between paternal exposure and SSR [73]. Levels of CB-153 and p, p’-DDE in serum were correlated with CAG repeats characteristic for androgen receptors. In the group with less than 20 CAG repeats, CB-153 was significantly related to decreased sperm number, assessed as total count and concentration, while p, p’-DDE was associated with sperm DNA fragmentation index (DFI) in the group with ≤21 CAG repeats. These results indicated that CAG repeats in androgen receptors might modify the effect of POPs on semen quality [74]. A large cross-sectional study on exposure to CB-153 and p, p’-DDE was conducted on men from Greenland, anglers from Sweden, and men from Kharkov in Ukraine and from Warsaw, Poland. They showed that sperm motility was inversely correlated with CB-153 concentration in Greenland and in Swedish anglers, while sperm concentration and morphology were not associated with CB-153 and p, p’-DDE levels in serum [75]. The same cohort was tested for X- and Y-chromosome-bearing sperm, and showed higher proportions of Y-chromosome sperm in Swedish and Greenlandic men, with higher serum levels of CB-153 compared to men from Warsaw and Kharkov. Log-transformed CB-153 and p, p’-DDE were also positively associated with Y-chromosome fractions in Swedish men. On the other hand, in a Polish cohort, CB-153 was negatively associated with the proportion of Y-chromosomes [76]. Since Inuits are highly exposed to POPs, their reproductive status and levels of CB-153 and p, p’-DDE were investigated. Inuits had low levels of DNA damage, tested by TUNEL assay, and their apoptosis markers were not associated with p, p’-DDE levels or CB-153 levels. These findings contrasted with a study on European males, in which exposure to CB-153 was associated with DNA integrity and levels of the anti-apoptotic protein Bcl-xL [77].

Urinary metabolites of non-persistent insecticides were also measured in men attending an infertility clinic in Poland. Lower sperm motility and higher DFI were associated with elevated concentrations of the chlorpyrifos metabolite 3,5,6-trichloro-2-pyridinol (TCPY). Furthermore, higher levels of morphologically abnormal sperm were associated with elevated levels of 1-naphtanol (1-N), a biomarker for the carbamate insecticide carbaryl [78]. A US case–control study measured non-persistent pesticide metabolites, which included the herbicides alachlor and atrazine as well as the insecticide diazinon, and men with higher serum levels of alachlor or diazinon were found to be more likely to have abnormal sperm parameters. These men came from agricultural regions of mid-Missouri [79,80].

**Table 2:** Epidemiologic studies estimating association between pesticide exposure and male infertility.

Study	Site	Design	Exposure	Duration of Exposure	Sample	Outcome	Results
Abell et al., 2000 [1]	Denmark	Cross-sectional	Self- and greenhouse owners reported workplace pesticide exposure	Short- and long-term	*n* = 122 healthy men from 30 ornamental flower greenhouses	Semen analysis and sex hormones	Median sperm concentration 40% lower in men with > 10 years vs. men with < 5 years’ experience. Age adjusted T/SHBG declined 1.9% (95%CI 0.4−3.4%) per year of work.
Aguilar-Garduno et al., 2012 [60]	Mexico	Longitudinal study	Urine DAP metabolites and serum *p,p’-*DDE	Long-term	*n* = 136 healthy industry workers	Semen analysis, sex hormones and PON1 activity	DEPT inversely assocaied with LH. Estradiol marginally significant positive trend with DEP and DEPT.
Bae et al., 2018 [73]	United States	Prospective cohort study	Serum OCPs (9), PBB (1), PBDEs (10) and PCBs (36)	Current	*n* = 235 men from couples undergoing fertility evaluation	Secondary sex ratio	Maternal PCB-128 and paternal HCB associated with female excess (RRs, 0.75 [95% CI, 0.60–0.94] and 0.81 [95% CI, 0.68–0.97]. Maternal mirex and paternal PCB 128 and *p,p’-*DDE associated with a male excess (RR range, 1.10–1.22). Only maternal mirex associated at *p* < 0.0009.
Celik-Ozenci et al., 2012 [59]	Turkey	Cross-sectional	Workplace abamectin exposure	Long-term	*n* = 20 exposed and 20 non-exposed men	Semen analysis, sperm maturity and sex hormones	Exposed group associated with decreased motility and increased sperm immaturity.
Cesaire Momo Tetsatsi et al., 2020 [50]	Cameroon	Descriptive retrospective study	Patient files	Historic	*n* = 379 men	Data on previous semen analysis	Farmers associated with the lowest quality spermogram.
Cremonese et al., 2017 [54]	Brazil	Cross-sectional	Self-reported application of pesticides	Long-term, maternal during pregnancy	*n* = 99 rural men and 36 urban men	Semen analysis, sex hormones and genital measurements	Rural men had poorer sperm morphology and count and lower LH. Maternal farming during pregnancy associated with larger AGD and TV.
Dhooge et al., 2007 [57]	Belgium	Cross-sectional	Reported consumption of self-grown vegetables	Long-term	*n* = 51 men	Semen analysis and sex hormones	Locally produced vegetables associated with lower free T, sperm concentration, morphology, LH and FSH decline (*p* = 0.04, *p* = 0.04, *p* = 0.002, *p* = 0.02*, p* = 0.08).
Dziewirska et al., 2019 [78]	Poland	Cross-sectional	Urinary 1N and TCPY	Current	*n* = 315 men with normal semen concentration, under 45 years of age	Semen analysis and DNA fragmentation	TCPY concentration associated with decreased motility, positive association with DFI. 1N negatively associated with normal morphology and positively with CASA parameters.
Giwercman et al., 2007 [74]	Europe	Cross-sectional	Serum cb-153 and *p,p’-*DDE	Long-term	*n* = 680 men from Greenland (188), Warsaw (167), Swedish fisherman (178), Kharkiv (147)	Semen analysis, sperm DNA fragmentation, CAG and GGN repeats in leukocytes	CB-153 group (*p* = 0.03) and CAG repeat category (*p* = 0.01) associated with sperm concentration and total count. *p,p’-*DDE associated with DFI (*p* = 0.01). Above vs. below median CB-153 associated with lower sperm concentration and total count for CAG < 20. DFI associated with *p,p’-*DDE for CAG ≤ 21
Haugen et al., 2011 [71]	Norway	Cross-sectional	Serum CB-153 and *p, p’*-DDE	Long-term	*n* = 197 men from southern or northern Norway	Semen analysis and sex hormones	Levels of *p, p’*-DDE, total and free T higher in south, FSH lower. CB-153 and SHBG associated in total, south and north cohorts (B = 0.12, *p* < 0.01; B = 0.13, *p* = 0.01; B = 0.12, *p* < 0.01)
Hauser et al., 2003 [70]	United States	Cross-sectional	Serum PCBs and *p, p’*-DDE	Current	*n* = 212 men undergoing fertility evaluation	Semen analysis	Dose–response relationship between PCB-138, sperm motility (OR = 1.00, 1.68, 2.35, *p*-value = 0.03) and morphology (OR = 1.00, 1.36, 2.53, *p* = 0.04)
Hu et al., 2020 [61]	China	Cross-sectional	Urinary pyrethroid metabolites	Current	*n* = 346 men undergoing fertility evaluation	Semen analysis	Negative association of 3PBA and morphology (ß = −2.12, 95%CI: −4.02 to −0.22), TDCCA and log transformed total count (ß = −0.09, 95%CI: −0.16 to −0.01); 4thQ 3PBA associated with lower sperm parameters and morphology (OR = 2.40, 95%CI: 1.26−4.54; OR = 3.08, 95%CI: 1.10−8.60).
Imai et al., 2014 [64]	Japan	Cross-sectional	Urinary 3-PBA	Current	*n* = 323 male university students	Semen analysis	No association between 3-PBA and semen quality
Ji et al., 2011 [62]	China	Cross-sectional	Urinary pyrethroid metabolites	Current	*n* = 240 men undergoing fertility evaluation	Semen analysis and DNA fragmentation	Inverse correlation between 3-PBA and sperm concentration (ß=−0.27, 95%CI: −0.41 to −0.12, *p* < 0.001). Positive correlation between 3-PBA and DFI (ß = 0.27, 95%CI: 0.15–0.39, *p* < 0.001).
Lwin et al., 2018 [51]	Myanmar	Cross-sectional	Self-reported application of pesticides	Long-term	*n* = 400	Semen analysis, sex hormones and plasma cholinesterase	Differences in seminal parameters and sex hormones (*p* < 0.05) between growing and nongrowing period. Cholinesterase levels significantly higher in growing periods (*p* < 0.05)
Luderer et al., 2013 [81]	United States	Nested case–control	Mothers of participants reported heptachlor epoxide exposure	Maternal, during pregnancy and lactation	*n* = 216 males and 183 females born in Oahu, Hawaii during 1981–1982	Semen analysis, sex hormones and onset of puberty	No strong association, weak association in males with higher FSH and LH concentrations, no dose–response relationship.
Meeker et al., 2008 [67]	United States	Cross-sectional	Urinary pyrethroid metabolites	Current	*n* = 207 men undergoing fertility evaluation	Semen analysis and sperm DNA damage	4thQ 3PBA associated with concentration reduction (95%CI: −37.1 to +2.6). 4thQ TDCCA associated with motility decline (95%CI: −26.2 to −4.8). 3- PBA and CDCCA associated with increased sperm DNA damage.
Miranda-Contresas et al., 2013 [55]	Venezuela	Cross-sectional	Self-reported workplace pesticide exposure	Long-term	*n* = 64 male farm workers and 35 healthy men, 18–52 years of age	Sperm analysis, DNA fragmentation, sex hormone analysis and cholinesterase activity	DFI negatively correlated with BuChE, sperm concentration, morphology and vitality in farm workers. No association with levels of Tt, PRL, FT4 and TSH. Tendency for increased LH and FSH in exposed workers.
Neghab et al., 2014 [53]	Iran	Cross-sectional	Self-reported application of pesticides	Long-term	*n* = 268 randomly selected married farm workers	Primary infertility	Primary infertility higher among farm workers (*p* < 0.05). Stillbirth and spontaneous abortion more common in wives of farm workers.
Pant et al., 2014 [69]	India	Case–control	Seminal levels of *p, p’*-DDE and lindane	Current	*n* = 278 men undergoing fertility evaluation (21–40 years of age)	Semen analysis, sperm mitochondrial status, ROS and SCSA	Correlation between seminal lindane, *p, p’*-DDE, concentration (r = −0.53, -0.48) and motility (r = −0.51, −0.37). Positive association between *p, p’*-DDE, lindane, ROS (r = 0.61, 0.53), lipid peroxidation (r = 0.58, 0.51) and MM dysfunction
Perry et al., 2016 [72]	United States	Cross-sectional	Adult serum (*n* = 90), cord blood and age-14 serum (*n* = 40) levels of *p, p’*-DDE and PCBs 118, 138, 153, 180.	Long-term	*n* = 90 men from Faroe Islands	X, Y, 18 chromosome disomy	3rd T vs. 1st T *p,p’*-DDE associated with XX18 (IRR = 1.52; 95%CI: 1.35, 1.72), XY18 (IRR = 1.40; 95%CI: 1.30, 1.51) and total disomy (IRR = 1.32; 95%CI: 1.25, 1.35). 2nd T YY & Σ_4_PCBs increased (IRR = 1.16; 95%CI: 1.03, 1.32), 3rd T decreased (IRR = 0.85; 95%CI: 0.74, 0.96). Similar in adults and those aged14.
Radwan et al., 2014 [66]	Poland	Cross-sectional	Urinary pyrethroid metabolites	Current	*n* = 334 men undergoing fertility evaluation	Semen analysis and sex hormones	Urinary pyrethroids associated with abnormal morphology (CDCCA, TDCCA, sum of pyrethroids) and decreased concentration, T level (TDCCA) and CASA parameters (LIN to 3-PBA and DBCA; VSL to VCL and DBCA).
Stronati et al., 2006 [77]	Europe	Cross-sectional	Serum CB-153 and *p, p’*-DDE	Long-term	*n* = 798 men from Greenland (201), Warsaw (198), Swedish fisherman (191), Kharkiv (208)	Sperm DNA fragmentation and apoptosis	CB-153 associated with altered sperm DNA integrity and Bcl-xL levels-No association for *p, p’*-DDE.
Swan et al., 2003 [80]	United States	Case–control	Serum pesticide and herbicide metabolites	Long-term	*n* = 50 cases and 36 controls	Semen analysis	Cases had higher levels of alachlor or IMPY (ORs = 30.0 and 16.7) and atrazine levels higher than LOD (OR = 11.3). 2,4-D and metolachlor associated with poor semen quality. Acetochlor levels lower in cases vs. controls (*p* = 0.04).
Swan et al., 2006 [79]	United States	Nested case–control	Serum non-persistent pesticide (8) metabolites	Current	*n* = 25 cases and 25 controls	Semen analysis	High levels of alachlor or diazinon (OR = 30.0, 16.7) and men with atrazine (OR = 11.3) over LOD significantly more among cases.
Tiido et al., 2006 [76]	Europe	Cross-sectional	Serum PCB-153 and *p, p’*-DDE	Long-term	*n* = 547 men from Greenland (157), Warsaw (121), Swedish fisherman (149), Kharkov (120)	Y:X ratio	Y-sperm associated with higher PCB-153 in Greenlandic and Swedish vs. Warsaw and Kharkov men. Log-transformed PCB-153 (*p* = 0.04) and *p, p’*-DDE (*p* < 0.001) associated with Y-fraction. Negatively associated PCB-153 and Y-chromosome in Polish cohort (*p* = 0.008)
Toft et al., 2006 [75]	Europe	Cross-sectional	Serum CB-153 and *p, p’*-DDE	Long-term	*n* = 763 men from Greenland (194), Warsaw (189), Swedish fisherman (185), Kharkov (195)	Semen analysis	In all regions, sperm motility associated with CB-153 blood concentration (ß = −3.6% per log unit CB-153 (ng/g lipid); 95%CI = −5.6 to −1.7). *p, p’*-DDE negatively associated with motility in Greenlandic population.
Toshima et al., 2012 [65]	Japan	Pilot study	Urinary metabolites of 5 phthalate diesters, pyrethroids, soy isoflavones and cadmium	Current	*n* = 42 men from couples undergoing fertility evaluation	Semen analysis	Weak positive correlation between concentration and cadmium (r = −0.316, *p* = 0.044). Negative correlation between concentration and daidzein (r = −0.292, *p* = 0.064). Equol correlated to motility (48.2 ± 21.0% in equol non-detectable group vs. 32.5 ± 16.8% in equol detectable, *p* = 0.013).
Young et al., 2013 [68]	United States	Cross-sectional	Urinary pyrethroids: 3-PBA, CDCCA and TDCCA	Current	*n* = 75 men undergoing fertility evaluation	X, Y and 18 chromosomes disomy	Sex chromosome disomies increased 7–30% in men above LOD vs. below LOD. YY18 disomy 1.28 times higher in 3-PBA group above LOD (95%CI: 1.15; 1.42). Reduced rate for XY18 and total disomy for 3-PBA (IRR = 0.82, 95% CI: 0.77; 0.87; IRR = 0.93; 95% CI: 0.87–0,97). No association for XX18 and 1818.
Yoshinaga et al., 2014 [63]	Japan	Cross-sectional	Urinary 3-PBA	Current	*n* = 322 male university students	Sex hormones	No association between 3-PBA and hormone levels.
Yucra et al., 2006 [56]	Peru	Cross-sectional	Self-reported workplace pesticide exposure	Long-term	*n* = 31 pesticide applicators and 80 non-exposed	Semen analysis and sex hormones	Volume (*p* = 0.02), motility grade A (*p* = 0.003), grade A+B (*p* = 0.002), normal morphology (*p* = 0.000) and zinc concentration (*p* = 0.02) lower, while pH (*p* = 0.003), liquefaction (*p* = 0.000), immature sperm *(p* = 0.01) and leukocytes (*p* = 0.000) higher in exposed men. T and LH levels lower (*p* = 0.001 and *p* = 0.02), T/LH significantly higher (*p* = 0.001) in exposed men.
Yucra et al., 2008 [58]	Peru	Cross-sectional	Urinary OP metabolites (6)	Long-term	*n* = 31 exposed and 31 non-exposed men	Semen analysis	DEDTP (*p* = 0.04) and DETP (*p* = 0.02) reflected occupational exposure. OP metabolites associated with lower volume and higher pH. Workplace and exposure time more strongly related to lower semen quality than urine OP metabolites.

Abbreviations—1N: 1-naphthol; 3-PBA: 3-phenoxybenzoic acid; AGD: androgenital distance; BuChE: butyrylcholinesterase; CASA: computer-aided sperm analysis; CB-153: 2,2′,4,4′,5,5′-hexachlorobiphenyl; CI: confidence interval; DBCA: dibromochloroacetic acid; DDE: dichlorodiphenyldichloroethylene; DEDTP: diethyldithiophosphate; DEPT: diethylthiophosphate; DFI: DNA fragmentation index; FSH: follicle-stimulating hormone; FT4: free thyroxine; HCB: hexachlorobenzene; IRR: indifence rate ratio; LH: luteneizing hormone; LIN: linearity; LOD: limit of detection; MM: mitochondrial membrane; OCP: organochlorine pesticides; OP: organophosphate pesticides; OR: odds ratio; PBB: polybrominated biphenyl; PBDE: polybrominated diphenyl ether; PCB: polychlorinated biphenyl; PRL: prolactin; ROS: reactive oxygen species; RR: realtive risk; SCSA: sperm chromatin structure assay; SHBG: sex hormone binding globulin; T: Testosterone; TCPY: 3,5,6-trichloro-2-pyridinol; TDCCA and CDCCA: trans- and cis-3-(2,2-Dichlorovinyl)-2,2-dimethylcy clopropane carboxylic acid; TSH: thyroid stimulatinf hormone; TV: testis volume; VCL: curvilinear velocity.

### 3.3. Transplacental Exposure Impact on Fertility

Transplacental exposure to pesticides is a very complex biological process whose final toxic or/and endocrine disrupting activity depends on the physicochemical properties of the substance or pesticide mixtures. Pesticides and their metabolites may interact synergistically or antagonistically. Using a human placental perfusion model for studying the pyrethroid cypermethrin and triazole fungicides propiconazole and bitertanol, it was shown that the placenta produces metabolites, thus exposing the mother and fetus to both the parent compounds and their metabolites [82]. The transplacental process is significantly affected by the lipid fraction, as lipophilic compounds can move across the placenta from maternal to fetal circulation. Accordingly, it is important to apply the lipid-adjusted ratio of concentrations in fetal and maternal circulation in risk assessments, as umbilical cord blood contains lower levels of lipids than maternal blood. The relationship of maternal and umbilical cord serum (or placenta) suggests that prenatal exposure to POPs may be estimated based on maternal serum pesticide concentrations [83,84]. The transplacental transfer of OCPs is a combination of simple diffusion and active transport [85]. Transplacental transfer depends on the physicochemical properties of pesticides such as polarity, molecular weight, lipophilicity and degree of chlorination or bromination, and may explain differences in maternal serum and cord blood pesticide levels [86]. The concentrations of organochlorine pesticides tend to be higher in mothers than in newborns and placental samples, except in the case of 4,4′-DDT, as fetal metabolizing and elimination mechanisms are less efficient than the mother’s [84]. Fetuses also have lower levels of detoxifying enzymes (e.g., paraoxonase or chlorpyrifos-oxonase) that deactivate organophosphates than adults [87], and are more susceptible to these exposures.

In a long-term Danish follow-up study of girls whose mothers were exposed very early in pregnancy to pesticides through their work as greenhouse workers, the mean onset of pubertal breast development was significantly earlier (8.9 years) in prenatally exposed girls, compared to daughters of unexposed mothers (10.4 years) and the Danish reference population. Transplacentally exposed girls had significantly higher serum androstenedione levels and lower AMH compared with unexposed participants, while levels of testosterone and estradiol did not differ between the groups [88]. Testicular volume and penile length were reduced in prenatally exposed sons of mothers occupationally exposed to pesticides. The effects were dose-related, although pituitary and testicular hormone serum concentrations did not differ between exposed and unexposed sons. Of 59 prenatally exposed boys, 8 had genital malformations, while there were no malformations among the unexposed boys [89]. However, in a similar study, the serum concentrations of sex hormone-binding globulin (SHBG) and the luteinizing hormone:testosterone ratio were increased compared to boys from unexposed mothers [90]. Occupational exposure to pesticides in both parents was a statistically significant risk for cryptorchidism, hypospadias and micropenis in newborns [91].

Organochlorine pesticide levels in the maternal blood, cord blood and placenta of mothers delivering small for gestational age babies were higher compared to mothers delivering appropriate for gestational age babies. In utero exposure to hexachlorocyclohexane (HCH) and other OCPs had a significant adverse effect on birth size. Following The Stockholm Convention (2001), HCH by itself is no longer produced or available on the market, but it is still present in the environment and in human populations [92,93]. The mother’s consumption of meat and milk significantly contributes to the levels of beta HCH in umbilical cord blood [94]. During fetal development, the female phenotype is the default condition, and male development requires testicular testosterone and AMH [95]. Thus, anti-androgenic OCP activities might be more severe in boys than estrogenic activities are in girls.

Although DDT has been banned in most parts of the world, it is still present in the environment and human exposure significantly varies between continents and countries [96]. In utero exposure to DDT is associated with reduced head circumference, crown–heel length, birth weight and birth length, which are independent of gestational age and/or preterm births. However, it has been suggested that this effect is detrimental [97].

Exposure to p,p’-DDE in utero is associated with shortened anal genital index among boys but not among girls, which may be caused by androgen deficiency after the reduction of transcriptional activity due to AR being blocked by p,p’-DDE in utero [98]. A significantly increased excess risk of urogenital malformations among the sons of exposed mothers was found in the presence of placental *o,p*’-DDT, *p,p*’-DDT, endosulfan-α, lindane and Mirex [99]. Maternal prenatal levels of *p*,*p*’-DDT were significantly inversely associated with testosterone levels, adjusted for Tanner stage [100], while there was no statistically significant association for p,p’-DDE [100,101].

In the daughters of mothers exposed to pesticides, the probability of a pregnancy fell by 32% per 10 microg/L p,p’-DDT in maternal serum, and increased 16% per 10 microg/L p,p’-DDE [102]. Both compounds are endocrine disruptors, which impact several mechanisms, such as p,p DEE induces aromatase and DDT suppresses androgen receptor-mediated activity [103,104].

Diuron (3-(3,4-di-chlorophenyl)-1,1-dimethylurea) is a herbicide banned in many parts of Europe, but due to its high stability and persistence, it is a contaminant of water in different areas of Europe and the US. Its toxic metabolite, 3-(3,4-dichlorophenyl)-1-methyl urea, crosses the human placenta and may result in fetotoxicity [105].

A disturbance of sex hormones in newborns was reported in association with maternal pesticide exposure in several studies. Significant reductions in estradiol, testosterone and the testosterone/E2 ratio were shown to be associated with dialkylphosphate, a metabolite that may disturb the development of the neuroendocrine axis, in newborns [106]. Similarly, sex hormone disturbances were detected for chlorpyrifos, which irreversibly inhibit CYP3A4 and the formation of 3-hydroxycarbofuran in human liver microsomes, causing te activation of testosterone metabolism and reductions in testosterone levels [107]. A gender-specific disturbance of sex hormones was reported for chlordanes, cis-HCB, heptachlor epoxide, Mirex and toxaphenes. Their levels in maternal blood were inversely associated with testosterone, SHBG and androstenedione-dehydroepiandrosterone in boys, while in girls, p,p’-DDT was inversely associated with the level of androstenedione-dehydroepiandrosterone [108,109].

An association between isolated hypospadias and the presence of the phenylurea herbicide isoproturon and the phenoxyherbicide 2-methyl-4-chlorophenoxyacetic acid in meconium has been detected [110].

It is important to stress that transplacental exposure to pesticides may disturb not only sex differentiation of the fetus, but also its sex-specific brain development. After crossing the placental barrier, pesticides pass through the blood–brain barrier of a fetus [111], which may impact behavior later in life [112], and as hypothesized for endocrine disruption, may affect gender identity in human populations [113].

### 3.4. Genetic Susceptibility to Pesticide Exposure

The majority of studies on interaction between genetic polymorphisms and exposure to pesticides have focused on cancer or neurodegenerative diseases, most often in case–control designs [114]. However, some studies of human paraoxonase 1 (PON1) polymorphisms and pesticide exposure have addressed other health effects, including effects on reproductive outcomes.

PON1 is a high-density lipoprotein (HDL)-associated esterase that hydrolyses a wide range of substrates, including some organophosphate insecticides. It also has antioxidant functions and protects lipoproteins from oxidative modifications [115]. PON1 is known to detoxify organophosphate insecticides by hydrolyzing the oxon-form of the parent compounds, and for most organophosphates the catalytic efficiency is higher among R-carriers than QQ-homozygotes [116]. Therefor QQ-homozygotes are more susceptible to acute toxic effects after high exposure to most organophosphates. However, for diazoxon (the active oxon-metabolite of diazinon), R-carriers have a higher catalytic rate, explaining their higher susceptibility towards high diazinone exposure among sheep dippers carrying the R-allele [117]. At lower exposure levels, the capacity to detoxify organophosphates is considered to be independent of the *PON1* Q192 genotype [118]. Thus, the higher susceptibility among R192-carriers reported in some of the studies above seems unrelated to the hydrolysis efficiency of the enzyme. The suggested mechanisms include lower anti-oxidative and anti-inflammatory efficiencies linked to the R-allele [115] causing R-carriers to be more vulnerable to adverse effects on, e.g., ovarian function and semen quality mediated by oxidative stress after pesticide exposure. A differential methylation profile in neuroendocrine signaling pathways was observed in DNA isolated from blood samples from children with the R-allele after prenatal pesticide exposure compared to exposed children with the QQ-genotype or unexposed children [119]. Whether similar *PON1*-related changes in DNA-methylation are induced in adults after pesticide exposure has, to our knowledge, not yet been investigated.

Several polymorphisms in *PON1* have been identified [119,120]. A common polymorphism in the coding sequence, a glutamine (Q)/arginine (R) substitute at position 192, affects both the catalytic capacity to detoxify organophosphates and the antioxidant function [115,121]. In a study from Mexico, women who had the RR genotype and were exposed to pesticides from floricultural work had a higher risk of having a baby with low birth weight than women with the QQ or QR genotype [122]. In another study, exposure to the organophosphate chlorpyrifos during pregnancy was associated with reduced head circumference in children at birth if the mothers had a low PON1 concentration, but no interaction was seen between exposure and maternal or child *PON1* genotype on head circumference, birth weight or birth length [123]. However, within the same birth cohort, negative associations between maternal urinary concentrations of organophosphate metabolites and cognitive development were seen at 12 months of age, if the mothers had the QR/RR genotype, although children of mothers with the QQ-genotype appeared to be more affected in later childhood [124]. In another study, low infant, but not maternal, PON1 enzyme activity was associated with shorter gestation and smaller head circumference at birth independently of maternal organophosphate exposure [125]. However, for infants with low PON1 activity and *PON1* -108TT and *PON1* 192QQ genotypes, prenatal organophosphate exposure was associated with shorter gestational agen. This finding might be related to lower expression levels of *PON1* in fetuses and neonates, implying higher susceptibility to pesticides [126,127]. In a genetic study of Caucasian, Caribbean Hispanic, and African American neonates and their mothers, three *PON1* promoter and two coding polymorphisms were investigated and for all genotypes the activity levels of the neonates were significantly lower than those of the mothers [128]. Another study on Latina mothers and their newborns showed that, the mean PON1 activity of the infants was fourfold lower than among the mothers [127]. In a Danish study among children of female greenhouse workers, prenatally pesticide-exposed children carrying the *PON1* 192R-allele had greater abdominal circumference, body fat content, body mass index (BMI) Z scores, blood pressure and serum concentrations of leptin and insulin growth factor-I at school age than unexposed children. The effects were associated with maternal pesticide exposure level during the first two months of pregnancy. For Q192 homozygote children, none of the variables were affected by prenatal pesticide exposure [129]. Furthermore, serum concentrations of leptin, glucagon and plasminogen activator inhibitor type-1 were enhanced in prenatally exposed children with the R-allele, also after adjusting for BMI [130]. The findings on body fat composition were still evident at adolescence (11–16 years) where dual X-ray absorptiometry (DXA), showed that android fat %, gynoid fat % and total fat % were positively associated with prenatal pesticide exposure in R-carriers. When maternal *PON1* genotype was also included, it was found that the associations were further strengthened if the mother also had an R-allele. In general, the associations were stronger in girls than in boys [131]. Thus, the potential impact of pesticide exposure during pregnancy might be modified by the genotype of both mother and fetus.

Regarding effects on male reproduction, a Chinese study on pesticide factory workers found that and exposed R192 homo/heterozygotes, but not Q192 homozygotes, had significantly lower sperm counts and fewer morphologically normal sperm compared to unexposed textile-factory workers. The results should be interpreted with caution because of a small sample size but the findings suggest that *PON1* genotype may modify the effect of organophosphate exposure on male fertility [132] which was also supported by another study among Mexican farmers [133]. Among farmers exposed to sheep dip, using the organophosphate pesticide diazinon as its active ingredient, self-reported ill individuals were more likely to be carriers of the R192 allele compared with those without symptoms [116,134]. A similar association was observed in a study of pesticide-exposed South African farmers [135,136].

## 4. Discussion

Our results show that multiple epidemiologic studies have reported statistically significant associations between reproductive disturbances and exposure to pesticides. However, most studies to date have focused on a single or limited set of pesticides, and there are few data available to assess the potential reproductive impacts of complex mixtures of the current, banned and new pesticides encountered by many populations, as well as potential interactions with other classes of endocrine disruptors.

While few relevant epidemiologic studies were captured by our search algorithm, the results were generally inconsistent, and as such inconclusive with respect to the associations of pesticide exposure with TTP and infertility. Even in large studies, the OCP results were inconsistent. Still, most studies used a retrospective assessment of TTP, which may introduce outcome misclassification, particularly for longer TTP [137], and enrolled women with ongoing pregnancies, excluding women with losses and without conception, who are possibly the most susceptible groups, which may bias the study results to null [138]. For non-persistent organophosphate, pyrethroid, and other pesticides, urinary metabolites offer a “snapshot” in time of recent exposure, and may thus misclassify exposure at critical biologic windows for reproductive health effects. In contrast, persistent OCPs and metabolites reflect long-term exposure, although physiologic changes may cause the misclassification of gestational exposure estimates in some women or introduce reverse causation [139], and non-fasting blood specimens may further confuse levels of lipophilic OCPs. Paternal exposures are also likely to play an important role in female fertility, and should be incorporated.

Genetic polymorphisms might play an important role in susceptibility to the adverse effects of pesticide exposure. For instance, PON1 levels differ significantly between individuals and between children and adults, a property that is highly relevant in the context of susceptibility to pesticides. Furthermore, *PON1* polymorphisms are also differently distributed between ethnic groups. Among Caucasians, a PON1 192R-allele frequency of approximately 25% has been reported, but in other populations the R-allele frequency was considerably higher (40–70%), particularly among South American and African populations, as well as being very high in Asian populations [140,141,142]. Thus, susceptibility to the adverse health effects of organophosphate insecticides is likely to be considerably different between different populations. Likewise, polymorphisms in other genes coding for pesticide-metabolizing enzymes (e.g., different cytochrome P450 (CYPs)) are differently distributed among populations and, although this is less studied, probably also affect susceptibility to the adverse reproductive health effects of pesticide exposure. Thus, genetic differences in vulnerability should be introduced into legislation.

Not only physiological, but also behavioral disturbances caused by the impact of pesticides on brain development may play a significant role for reduced fertility [143].

The potential of publication bias is recognized as errors in search strategies can impact the quality and validity of systematic reviews [144]. Our search strategy was carefully considered to help ensure the adequate coverage of the literature, and that no key articles were overlooked. The review was therefore reliant on the methodology employed, but also the published literature that is available. Our review focused on articles published in the English language. Given that others have cited that over roughly 80% of all the journals indexed in Scopus are published in English (van Weijen, 2012; https://www.researchtrends.com/issue-31-november-2012/the-language-of-future-scientific-communication/ accessed on 1 June 2021) [145], and with more than 90% of the indexed scientific articles in the natural sciences being published in this language [146], it is considered that our review provides good coverage of the relevant publications. In our review, as part of the inclusion criteria, we focused on identifying studies where there had been self-reported occupational exposure and/or measured levels of metabolites in blood or urine. Both direct (e.g., biomonitoring) and indirect (e.g., self-reported exposures) exposure assessment methods (EAMs) are intrinsically associated with degrees of exposure misclassification, which might lead to conflicting study results [147]. The Improving Exposure Assessment Methodologies for Epidemiological Studies on Pesticides (IMPRESS) project aims to improve the understanding of the performance of pesticide EAMs used in previous epidemiological investigations [148], and it is considered that the future outputs of this will prove valuable when interpreting such studies.

## 5. Conclusions

In conclusion, our results show that the most frequently investigated pesticides with reported associations with human fertility are OCPs and their mixtures. Additionally, the urinary metabolites of pyrethroids were also investigated, which were often applied together with OCPs. 

At present, fertility evaluations are not routinely incorporated into occupational health surveillance programs. However, our review of the literature suggests that this is crucial. Despite the fact that collecting data on fertility may cause discomfort in workers, or simply a refusal to respond, the introduction of biomarkers such as sex hormone levels could show the trend of exposure and the expected biological effect.

Future studies should also incorporate pesticide exposure in both partners, as available studies very rarely give holistic insights into family-level exposure.
